# Anti-neuroinflammation ameliorates systemic inflammation-induced mitochondrial DNA impairment in the nucleus of the solitary tract and cardiovascular reflex dysfunction

**DOI:** 10.1186/s12974-019-1623-0

**Published:** 2019-11-15

**Authors:** Mu-Hui Fu, I-Chun Chen, Chou-Hwei Lee, Chih-Wei Wu, Yu-Chi Lee, Yu Chih Kung, Chun-Ying Hung, Kay L. H. Wu

**Affiliations:** 1 Department of Neurology, Kaohsiung Chang Gung Memorial Hospital, Kaohsiung, Taiwan Republic of China; 2 Institute for Translational Research in Biomedicine, Kaohsiung Chang Gung Memorial Hospital, Kaohsiung, 83301 Taiwan Republic of China; 3 Master of Science Program in Health Care, Department of Nursing, Meiho University, Neipu Township, Republic of China; 4 Department of Nursing, Meiho University, Neipu Township, Taiwan, Republic of China; 5 Department of Senior Citizen Services, National Tainan Institute of Nursing, Tainan, 700 Taiwan Republic of China

**Keywords:** Systemic inflammation, Neuroinflammation, Proinflammatory cytokines, mtDNA damage, mtDNA repairment, Baroreflex, NTS

## Abstract

**Background:**

Decreased heart rate variability (HRV) leads to cardiovascular diseases and increased mortality in clinical studies. However, the underlying mechanisms are still inconclusive. Systemic inflammation-induced neuroinflammation is known to impair the autonomic center of cardiovascular regulation. The dynamic stability of blood pressure and heart rate (HR) is regulated by modulation of the reciprocal responses of sympathetic and parasympathetic tone by the baroreflex, which is controlled by the nucleus of the solitary tract (NTS).

**Methods:**

Systemic inflammation was induced by *E. coli* lipopolysaccharide (LPS, 1.2 mg/kg/day, 7 days) peritoneal infusion via an osmotic minipump in normotensive Sprague-Dawley rats. Systolic blood pressure (SBP) and HR were measured by femoral artery cannulation and recorded on a polygraph under anesthesia. The low-frequency (LF; 0.25–0.8 Hz) and high-frequency (HF; 0.8–2.4 Hz) components of SBP were adopted as the indices for sympathetic vasomotor tone and parasympathetic vasomotor tone, while the baroreflex effectiveness index (BEI) was adopted from the analysis of SBP and pulse interval (PI). The plasma levels of proinflammatory cytokines and mitochondrial DNA (mtDNA) oxidative damage were analyzed by ELISA. Protein expression was evaluated by Western blot. The distribution of oxidative mtDNA was probed by immunofluorescence. Pharmacological agents were delivered via infusion into the cisterna magna with an osmotic minipump.

**Results:**

The suppression of baroreflex sensitivity was concurrent with increased SBP and decreased HR. Neuroinflammatory factors, including TNF-α, CD11b, and Iba-1, were detected in the NTS of the LPS group. Moreover, indices of mtDNA damage, including 8-OHdG and γ-H2AX, were significantly increased in neuronal mitochondria. Pentoxifylline or minocycline intracisternal (IC) infusion effectively prevented mtDNA damage, suggesting that cytokine and microglial activation contributed to mtDNA damage. Synchronically, baroreflex sensitivity was effectively protected, and the elevated blood pressure was significantly relieved. In addition, the mtDNA repair mechanism was significantly enhanced by pentoxifylline or minocycline.

**Conclusion:**

These results suggest that neuronal mtDNA damage in the NTS induced by neuroinflammation could be the core factor in deteriorating baroreflex desensitization and subsequent cardiovascular dysfunction. Therefore, the enhancement of base excision repair (BER) signaling in mitochondria could be a potential therapeutic strategy for cardiovascular reflex dysregulation.

## Background

A previous clinical study reported that decreased heart rate variability (HRV) can lead to cardiovascular disease and increased mortality, particularly sudden death [1]. However, the underlying mechanisms are still inconclusive. Systemic inflammation has been demonstrated to trigger neuroinflammation in accumulating studies. Both animal and human studies have documented a surge in proinflammatory cytokines, including tumor necrosis factor α (TNF-α), interleukin-6 (IL-6) and IL-1β, and activation of microglia in the central nervous system during systemic inflammation [2, 3]. The accumulation of cytokines and microglial activation, which are the landmarks of neuroinflammation, play important roles in suppressing neuronal function via oxidative stress [4]. Nevertheless, the detailed mechanisms of impaired neuronal function remain elusive.

The maintenance of blood pressure (BP) in response to environmental changes is critical for individual survival when uncontrolled BP could lead to lethal diseases, such as severe hypertension. Elevated BP activates baroreceptors in the carotid sinus and in the aortic arch. These baroreceptor sensory inputs send bursts to sensory neurons in the nucleus of the solitary tract (NTS), resulting in inhibition of sympathetic drive and excitation of vagal tone to reverse the BP; this mechanism is known as the baroreflex (BRR) [5]. Baroreflex sensitivity (BRS, or baroreflex effectiveness index, BEI), defined as the change in interbeat interval in milliseconds per unit change in BP, is an important tool for evaluating NTS function in balancing BP [5].

Pathological damage to the NTS suppresses BRS, raises BP, and may even cause sudden death [6, 7]. Systemic inflammation induces increased levels of proinflammatory cytokines in the NTS [8]. These cytokines are capable of influencing the activity of NTS neurons [9] and can lead to BRR desensitization. A previous study suggested that cytokine-induced oxidative stress in neurons dampens neuronal activity [2]. However, the underlying mechanism is largely unknown.

Mitochondria are the only cellular organelle with self-DNA (e.g., mitochondrial DNA, mtDNA) other than the nucleus for replication and protein synthesis, and this mtDNA serves to maintain and renew the mitochondrial mass. Endogenous reactive oxygen species (ROS) can cause a large degree of DNA damage [10]. Mitochondrial oxidative phosphorylation for cellular energy produces a lethal side product, ROS. Without histone protection, mtDNA, which are supercoiled and closed circular molecules, is more vulnerable than nuclear DNA (nDNA) to oxidative stress [11, 12]. mtDNA damage and reduced bioenergetics have been observed in chronic neuroinflammatory disorders [13]. 8-hydroxy-2′-deoxyguanosine (8-OHdG), an oxidized form of guanine, is the main oxidative DNA damage product that results from ROS [14]. mtDNA damage by oxidative stress contributes to mtDNA loss [15], while loss of mtDNA in the human brain results in mitochondrial dysfunction and subsequent neuronal disorder [16].

In response to mtDNA damage, DNA repair mechanisms are essential for the maintenance of proper cellular function and survival. The base excision repair (BER), which excises and replaces damaged (e.g., 8-OHdG) bases, is the only DNA repair pathway in mitochondria [17], while several systems are involved in nuclear DNA repair mechanisms [18]. The BER mechanism comprises five common steps to repair mtDNA damage: first, the damaged base is recognized and removed by a DNA glycosylase (such as uracil-DNA glycosylase, UNG); second, a basic site is incised by an apurinic/apyrimidinic endonuclease (APE1); third, the remaining sugar fragment is removed by a lyase or phosphodiesterase; fourth, the gap is filled by a DNA polymerase; and fifth, the nick is sealed by a DNA ligase [19]. In addition, the DNA repair protein RAD51 homolog 1 (RAD51) has been documented to repair mitochondrial DNA breaks [20]. Whether the oxidative stress-damaged mtDNA has been properly repaired may determine the fate of those stressed neurons under inflammation.

A rodent model of sustained, low-grade systemic inflammation [2] was employed in the present study. We investigated whether neuroinflammation enhanced mtDNA oxidative damage in the NTS to dysregulate the baroreflex and manifest raised BP under systemic inflammation.

## Methods

### Animals

Adult, male Sprague-Dawley (SD) rats (8 weeks old, 250–280 g, *n* = 96) were purchased from BioLASCO Taiwan Co., Ltd. SD rats were housed in an AAALAC International-accredited animal facility and maintained under temperature control (21 ± 0.5 °C) and a 12-h light-dark cycle (lights on during 05:00–17:00). Standard chow and tap water were provided ad libitum. Animals were acclimatized for 7 days before experimental manipulations. All experimental procedures were carried out in compliance with the guidelines of the institutional animal care and use committee (IACUC) of Kaohsiung Chang Gung Memorial Hospital (IACUC number 2016090502).

### Induction of systemic inflammation by an osmotic minipump

Continuous infusion of *Escherichia coli* lipopolysaccharide (LPS; serotype 026:B6; Sigma-Aldrich, St. Louis, MO) (1.2 mg/kg/day dissolved in saline, 7 days) was conducted to establish a rodent model of transient systemic inflammation [2]. Animals were anesthetized with sodium pentobarbital (50 mg/kg, IP) to place an osmotic minipump (Alzet 1007D; Durect Co., Cupertino, CA) in the peritoneal cavity. Control animals received saline-filled osmotic minipumps. After implantation and suturing, the animals received intramuscular procaine penicillin (1000 IU) injection. The body temperature of the operated animals was maintained at 37 °C with a heating pad until the animals recovered from anesthesia.

### Measurement of systemic arterial pressure and heart rate

Baseline systolic blood pressure (SBP) and heart rate (HR) were recorded for 3 days in conscious rats using the noninvasive tail-cuff method based on electrosphygmomanometry (MK-2000; Momuroki Kikai Co., Japan). Only rats with similar levels of SBP and HR were used for the study. Then, implantation of an osmotic minipump for IP infusion of LPS (1.2 mg/kg/day) or saline for 7 days was conducted. SBP and HR were measured in rats under sodium pentobarbital (50 mg/kg, IP) anesthesia. Each animal was placed on a thermostatically controlled pad to maintain a rectal temperature of 37 ± 0.5 °C. SBP and HR were measured by femoral artery cannulation and recorded on a polygraph (Notocord, Le Pecq, France) [4]. Baseline SBP was recorded for 15 min.

### Power spectral analysis of arterial pressure signals

Continuous and real-time autospectral analysis (Notocord, Le Pecq, France) of SBP signals based on fast Fourier transform was employed to detect temporal fluctuations in the low-frequency (LF; 0.25–0.8 Hz) component, which was the experimental index for sympathetic vasomotor tone; the high-frequency (HF; 0.8–2.4 Hz) component, which was the experimental index for parasympathetic vasomotor tone; and the BEI. The SBP spectra and power density of the low- and high-frequency components were displayed continuously during the experiment, alongside SBP and HR, in a real-time manner. The LF/HF ratio was used as the experimental index as the balance between sympathetic and parasympathetic activity [21].

### Intracisternal infusion of test agents by osmotic minipump

After LPS implantation, some animals underwent an additional implantation of a micro-osmotic pump (model 1007D, ALZET) with the aid of a brain infusion kit (brain infusion kit 2, ALZET) for intracisternal (IC) infusion of the test agent. The dura mater between the foramen magnum and the C1 lamina was exposed by a midline dorsal neck incision followed by perforation with a 24-gauge steel needle. Then, a PE-10 catheter (2.5 mm, Clay Adams, Sparks, MD) was inserted into the cisterna magna. The catheter was sealed to the dura with tissue glue, and the incision was closed with layered sutures. The outer end of the catheter was connected to an osmotic minipump, which was placed under the skin in the neck region. The test agents used for IC infusion included an anti-inflammatory drug, pentoxifylline (PTX; 30 nmol/μL/h, Sigma-Aldrich), or an inhibitor of microglial activation, minocycline (mino; 9 nmol/μL/h; Sigma-Aldrich). These agents were continuously infused into the cisterna magna for 7 days. The doses of the test agents were determined in pilot experiments. Control infusion of sterile saline served as the volume and vehicle control.

### Analysis of plasma inflammatory markers

Six milliliters of blood was collected from the heart and mixed with 6 mL Minicollect® tripotassium EDTA (Greiner bio-one, Monroe, NC). After centrifugation at 2000×*g* for 30 min at 4 °C, the supernatant was collected for quantitative analysis of TNF-α, IL-1β, or IL-6 by sandwich enzyme-linked immunosorbent assay (ELISA) (R&D Systems, Inc., MN, USA) according to the manufacturer’s instructions. In brief, samples or standards mixed with diluent were loaded onto a 96-well plate to react with biotin-conjugate for 2 h at room temperature. After washing, streptavidin-HRP was applied to each well, and the samples were incubated for 1 h at room temperature, followed by washing and TMB substrate for 10 min. The colorimetric reaction product for individual proinflammatory factors was measured at 450 nm using a microplate reader (Eppendorf, London, UK). The concentrations of TNF-α, IL-1β, or IL-6, expressed in picograms per milliliter (pg/mL), were determined from the regression line for the standards incubated under the same conditions in each assay. All assays were performed in triplicate.

### Collection of tissue samples from NTS

After experimental treatment, rats were sacrificed with an overdose of pentobarbital sodium (100 mg/kg, IP) and perfused intracardially with 37 °C saline. The brain was rapidly removed and immediately frozen in ice-cold saline. The medulla oblongata covering the NTS was blocked between 0.5 mm cranial and 0.5 mm to the calamus scriptorius, as adopted from the atlas of Watson and Paxinos [22]. Both sides of the NTS were collected by micropunches with a 1-mm inner diameter stainless-steel burr [2]. Medullary tissues collected were stored at − 80 °C for subsequent protein analysis.

### Isolation of cytosolic and mitochondrial fractions

The NTS homogenate obtained from a Dounce grinder with a loose pestle was centrifuged at 2000×*g* for 10 min. The supernatant was obtained for further centrifugation at 13,500 for 30 min. The supernatant was obtained as cytosolic protein, and the pellet was resuspended in ice-cold isolation buffer (320 mM sucrose, 1 mM EDTA, 10 mM Tris-HCl, pH 7.4) as the mitochondrial fraction. The mitochondrial layer was extracted by discontinuous Percoll gradient centrifugation [23]. Isolation of NTS mitochondria was performed at 4 °C and completed within 2 h of tissue collection. This procedure yields 10–15% of the total mitochondria and enriches the mitochondrial fraction by at least tenfold compared with tissue homogenates [24]. The purity of the cytosol, mitochondrial-rich fraction and nuclei was verified by the expression of GAPDH and prohibitin. The concentration of protein in each fraction was estimated by the Bradford method with a protein assay kit (Bio-Rad).

### Nuclear protein extraction

The collected NTS was homogenized with a Dounce grinder with a loose pestle in ice extraction buffer (BioVision, Inc., CA, USA). The homogenate was centrifuged at 13,500×*g* for 10 min. Then, the pellet was resuspended in nuclear extraction buffer (BioVision, Inc.), vortexed for 15 s followed by 10 min on ice, which was repeated for 40 min, and finally, centrifuged at 14,000×*g* for 30 min at 4 °C. The supernatant was saved as the nuclear protein. The purity of the nuclear-rich fraction was verified by the expression of TATA box binding protein (TBP). Protein concentration was determined by the Bradford assay (Bio-Rad).

### Western blot analysis

Cytosolic protein from NTS samples was used to detect the expression of TNF-α, IL-1β, IL-6, ionized calcium-binding adapter molecule 1 (Iba-1) and cluster of differentiation molecule 11b (CD11b), which are markers of proinflammatory cytokines and microglial activation. GAPDH was applied as the internal control to normalize the expression levels of these proteins. The mitochondrial protein extracted from the NTS samples was used to detect factors of mitochondrial DNA damage and repairment, including 8-OHdG, γ-H2AX, APE1, RAD51, mitochondrial fission 1 protein (FIS1), phospho-dynamin related protein 1 (p-Drp1), mitofusin 1 (MFN1), and MFN2. The levels of these factors were normalized to the level of prohibitin. Protein samples were subjected to 10% SDS-PAGE polyacrylamide gel electrophoresis under denaturing conditions. Proteins were transferred onto PVDF transfer membranes (PerkinElmer Life Sciences, Waltham, MA) for 1.5 h at 4 °C using a Bio-Rad miniprotein-III wet transfer unit (Bio-Rad). The transfer membranes were then incubated with a blocking solution (5% nonfat dried milk dissolved in Tris-buffered saline–Tween buffer (pH 7.6, 10 mM Tris-HCl, 150 mM NaCl, and 0.1% Tween 20) for 1 h at room temperature.

The primary antisera used included mouse polyclonal antiserum against TNF-α (1;1000, Abcam, UK), IL-1β (1;1000, Abcam, UK), IL-6 (1;1000, Abcam, UK), Iba-1 (1;1000, Wako, Japan) and CD11b (1;1000, Abcam, UK), γ-H2AX (1:500, Abcam, UK), UNG (1:1000; Abcam, UK), APE1 (1:1000; Abcam, UK), RAD51 (1:1000, Santa Cruz Biotechnology, Inc., CA, USA), FIS1 (1:1000, Sigma-Aldrich, MO, USA), p-Drp1 (1:1000, Cell Signaling Technology, Inc., MA, USA), MFN1 (1:1000, Abcam, Cambridge, UK), MFN2 (1:1000, Cell Signaling Technology, Inc.), GAPDH (1:50000, Millipore, MA, USA) and prohibitin (1:10000; Thermo Fisher Scientific, MA, USA). Membranes were washed three times with TBS-t buffer followed by the secondary antibodies (1:20000; Jackson ImmunoResearch, West Grove, PA) for 2 h. Specific antibody-antigen complexes were detected using enhanced chemiluminescence. The amount of detected protein was quantified by ImageJ software (NIH, USA) and was expressed as the ratio to prohibitin protein.

### Measurement of mitochondrial 8-OHdG

NTS mitochondria were collected for quantitative analysis of 8-OHdG by sandwich ELISA (Bender MedSystems, Vienna, Austria) according to the manufacturer’s instructions. In brief, samples or standards mixed with diluent were loaded onto a 96-well plate to react with biotin-conjugate for 2 h at room temperature. After washing, streptavidin-HRP was applied to each well, and samples were incubated for 1 h at room temperature, followed by washing and TMB substrate for 10 min. The reaction was stopped by adding the Stop solution. The colorimetric reaction product for *8-OHdG* was measured at 450 nm using a microplate reader (ThermoScientific). The concentration of *8-OHdG* in NTS, expressed in nanograms per microliter (ng/μL), was determined from the regression line of the standards incubated under the same conditions in each assay. All assays were performed in triplicate.

### Immunofluorescence

For morphological analyses, brainstems were removed and postfixed in 4% paraformaldehyde for 72 h at 4 °C after saline perfusion. Next, the samples were cryoprotected in 30% sucrose solution and stored at − 20 °C for further immunohistochemical staining. For immunostaining, brainstems were sliced with a freezing microtome at 30 μm. Samples were collected in cryoprotectant (30% ethylene glycol, 20% glycerol, 50 mmol/L sodium phosphate buffer, pH 7.4). After washing with PBS buffer and permeabilization with 0.1% Triton X 100 in 0.1% sodium citrate, the 4% paraformaldehyde fixed brain slices were incubated with rabbit anti-8-hydroxyguanosine (1: 200, Abcam) in 10% goat serum PBS buffer. The slices were then incubated with green fluorescent-conjugated secondary antibody (Thermo Fisher Scientific Inc.) for 1 h. After washing, the slices were incubated with rabbit anti-NeuN (1: 500, Millipore) in 10% goat serum PBS buffer followed by a red fluorescent-conjugated secondary antibody (Thermo Fisher Scientific Inc.) for another 1 h. DAPI was used as the blue nuclear stain. The expression and distribution of fluorescent signals were detected under an FV10i Confocal Laser Scanning Microscope (Olympus, Tokyo, Japan).

### Immunohistochemistry

A portion of slides from each group were selected for microglial identification by rabbit anti-Iba-1 (1: 1000; Wako Chemicals USA, Inc., VA, USA). Brain specimens were incubated with the appropriate peroxidase-conjugated secondary antibody (Vector, Burlingame, CA, USA) and an avidin–biotin peroxidase (Vector) using 3,3′diaminobenzidine as the substrate in black color. After counterstaining with neutral red for the cytosol, images were observed with an Olympus BX50 DIC light microscope (Olympus, Tokyo, Japan).

### Statistical analysis

Data are expressed as the means ± SEM. The statistical software GraphPad Prism 5.0 (La Jolla, CA) was used for data analysis. Student’s *t* test or one-way analysis of variance with repeated measures were used, as appropriate, to assess group means, followed by Tukey’s multiple range test for post hoc assessment of individual means. Differences corresponding to a *P value < 0.05 vs. the control (C) group and a ^#^*P* value < 0.05 vs. The LPS group were considered statistically significant.

## Results

### Increases in plasma proinflammatory cytokines induced by continuous LPS peritoneal infusion

To evaluate the level of systemic inflammation under peripheral infusion of LPS (1.2 mg/kg/day) for 7 days, the plasma levels of proinflammatory cytokines, including TNF-α, IL-1β and IL-6, were detected by ELISA. Compared with saline infusion, peritoneal infusion with LPS for 7 days significantly increased the plasma levels of TNF-α and IL-1β (Table 1). Intracisternal infusion with pentoxifylline (PTX, 30 nmol/μL/h) or minocycline (mino, 9 nmol/μL/h) to inhibit cytokine expression or microglial activation, respectively, did not reduce the plasma levels of cytokines induced by LPS.

**Table 1:** The plasma levels of TNF-α, IL-1β and IL-6 in the control, LPS and LPS with pentoxifylline or minocycline treatment groups

(pg/mL)	C	LPS	LPS+PTX	LPS+mino
*TNF-α*	7.92 ± 1.6	29.21 ± 3.1**	25.46 ± 9.6*	19.35 ± 3.8
*IL-1β*	19.08 ± 2.2	49.39 ± 4.1**	37.33 ± 9.4	38.20 ± 4.4*
*IL-6*	2659 ± 62.5	2946 ± 3.0	3176 ± 168.8	2980 ± 228.8

Values are the mean ± SEM of 12 to 16 animals in each group. *P < 0.05, **P < 0.01 versus the vehicle control group in the post hoc Tukey’s multiple range analysis. *C* control, *L* LPS, lipopolysaccharides, *PTX* pentoxifylline, *mino* minocycline

### Baroreflex dysregulation and aberrant hemodynamics concurrent with increases in proinflammatory cytokines induced by systemic LPS infusion

**Fig. 1:**
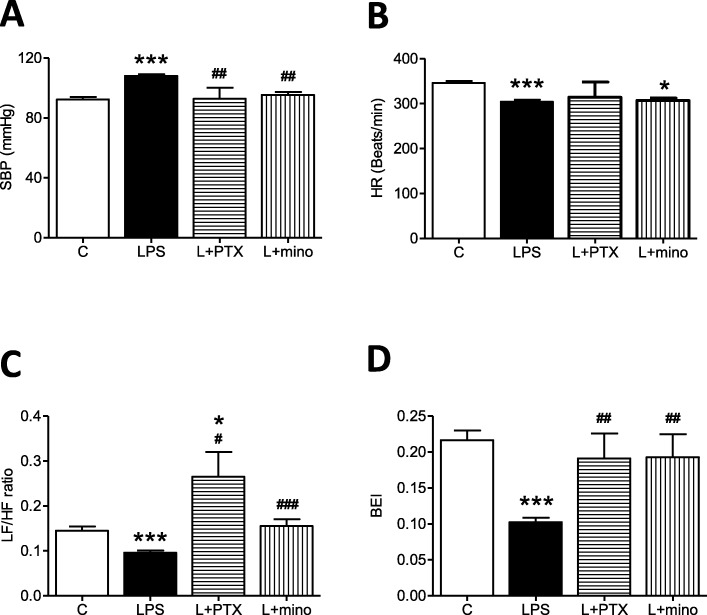
Peritoneal infusion with LPS disturbed blood pressure, heart rate, and baroreflex sensitivity in young Sprague-Dawley (SD) rats. The changes in **a** systolic blood pressure (SBP), **b** heart rate (HR), **c** the ratio of the power density of the low-frequency (LF) component to high-frequency (HF) component of the systolic blood pressure spectrum, and **d** baroreflex effectiveness index (BEI) after peritoneal infusion with saline or LPS for 7 days with additional intracisternal (IC) infusion of saline, PTX or mino. Values are the mean ± SEM of 12 to 15 animals in each group. ***P < 0.001 vs the control (C) group, and ^*#*^*P* < 0.05, ^*##*^*P* < 0.01, ^*###*^*P* < 0.001 vs the LPS group according to post hoc Tukey’s multiple range analysis. C, control, infused with saline; L, LPS, lipopolysaccharides; mino, minocycline; PTX, pentoxifylline

To evaluate whether the baroreflex sensitivity and hemodynamics were impaired by systemic inflammation, SBP, HR, LF (an index of sympathetic activity), HF (an index of parasympathetic activity) and BEI (an index of baroreflex effectiveness) were measured after 7 days of peritoneal LPS infusion. Compared with saline infusion, peripheral infusion of LPS for 7 days resulted in a significant increase in SBP (Fig. 1a) concurrent with the decreases in HR (Fig. 1b), the LF/HF ratio (Fig. 1c) and baroreflex effectiveness (Fig. 1d). PTX central infusion effectively reversed the changes in SBP, the LF/HF ratio and BEI. Moreover, minocycline infusion significantly reversed the elevation in SBP and the suppressed the LF/HF ratio and BEI.

### Neuroinflammation in NTS induced by peritoneal LPS infusion

**Fig. 2:**
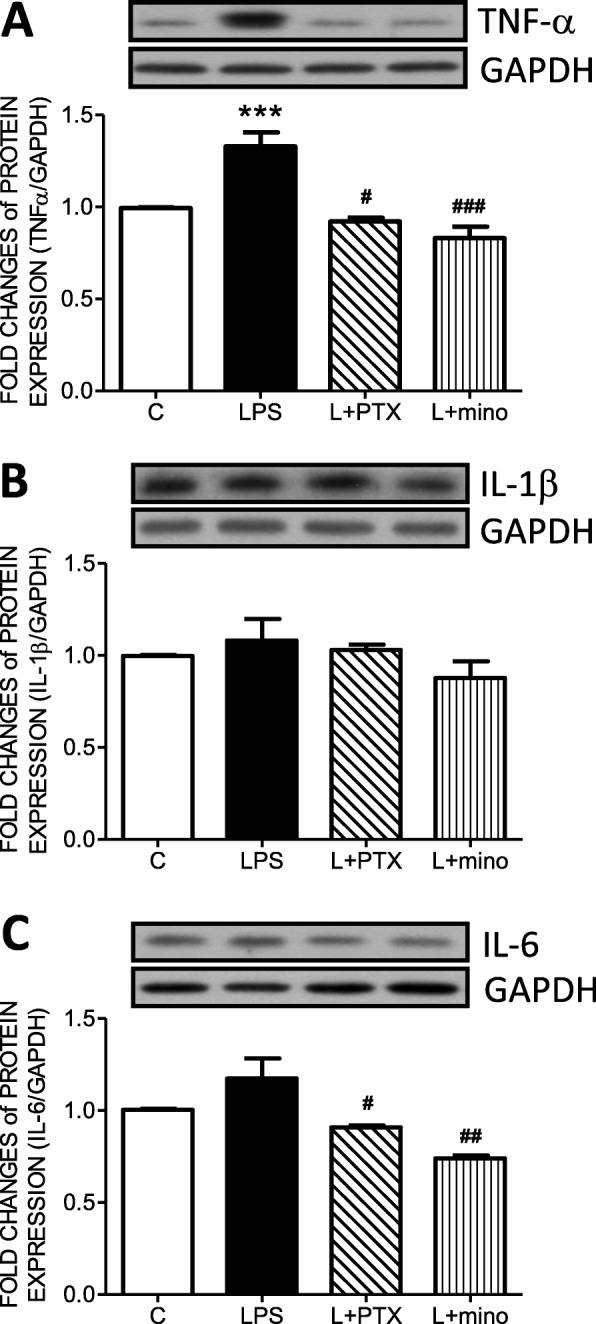
Peritoneal infusion with LPS increased the accumulation of proinflammatory cytokines in the nucleus of the solitary tract (NTS). Representative gels (inset) and densitometric analysis from Western blot showed the total protein expression of (**a**) TNF-α, (**b**) IL-β, and (**c**) IL-6 in the NTS after peritoneal infusion with saline or LPS for 7 days with additional intracisternal (IC) infusion of saline, PTX, or mino. GAPDH was used as the internal control for total protein expression. Values are the mean ± SEM of 6 to 8 animals in each group. *P < 0.05, ***P < 0.001 vs the control (C) group and ^*#*^*P* < 0.05, ^*##*^*P* < 0.01, ^*###*^*P* < 0.001 vs the LPS group according to post hoc Tukey’s multiple range analysis. C, control, infused with saline; GAPDH, glyceraldehyde 3-phosphate dehydrogenase; L, LPS, lipopolysaccharides; mino, minocycline; PTX, pentoxifylline

To evaluate whether LPS peritoneal infusion induced neuroinflammation in the NTS, the levels of proinflammatory cytokines, including TNF-α, IL-1β, and IL-6, were detected by Western blot. The results indicated that TNF-α (Fig. 2a) was significantly upregulated in the LPS group compared with the control group, while the levels of IL-1β (Fig. 2b) and IL-6 (Fig. 2c) showed no significant changes between groups. PTX central infusion effectively decreased the levels of TNF-α and IL-6. Moreover, minocycline infusion significantly decreased the levels of TNF-α and IL-6.

**Fig. 3:**
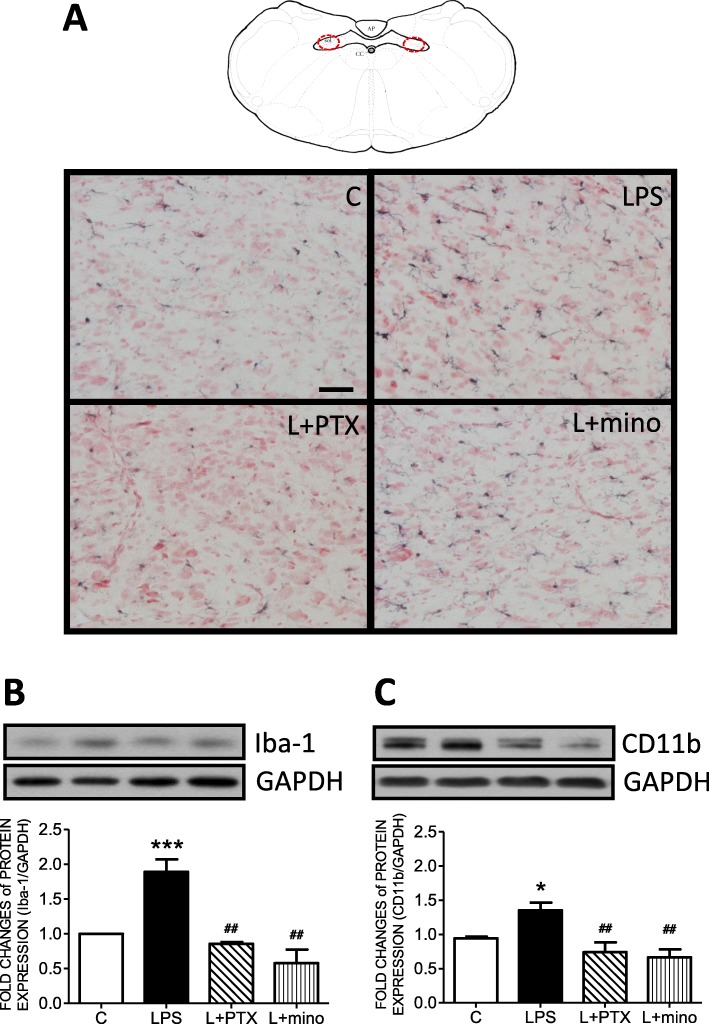
Peritoneal infusion with LPS increased microglial activation in the nucleus of the solitary tract (NTS). **a** A representative schematic diagram and microscopy images of Iba-1 immunohistochemistry staining in the NTS and representative gels (inset) and densitometric analysis of Western blot showing the total protein expression of **b** Iba-1 and **c** CD11b in the NTS after peritoneal infusion with saline or LPS for 7 days with additional intracisternal (IC) infusion of saline, PTX or mino. GAPDH was used as the internal control for total protein expression. Neutral red was used as the counterstain. Values are the mean ± SEM of 6 to 8 animals in each group. *P < 0.05, ***P < 0.001 vs the control (C) group and ^*##*^*P* < 0.01 vs the LPS group according to post hoc Tukey’s multiple range analysis. AP, area postrema; C, control, infused with saline; cc, central canal; GAPDH, glyceraldehyde 3-phosphate dehydrogenase; Iba-1, ionized calcium-binding adapter molecule 1; L, LPS, lipopolysaccharides; mino, minocycline; PTX, pentoxifylline; sol, solitary tract. Scale bar, 50 μm

To further delineate whether microglia were activated, microglial morphology was detected by immunohistochemical staining with Iba-1. Representative images from the area of the NTS denoted by the red circle further indicated an increase in microglial ramification in the LPS group compared with the control (C) group (Fig. 3a). Semiquantification with Western blotting indicated that the expression levels of Iba-1 (Fig. 3b) and CD 11b (Fig. 3c) were upregulated in the LPS group compared with the control group.

To further delineate the role of cytokines in microglial activation, PTX was centrally infused to inhibit cytokine expression in the NTS. The PTX-infused group exhibited fewer morphological changes and less protein expression of ionized calcium-binding adapter molecule 1 (Iba-1) than the LPS group. Additionally, the expression of CD11b, an index of microglial activation, was significantly suppressed in the LPS with PTX (L+PTX) group (Fig. 3).

Furthermore, minocycline was centrally infused to inhibit microglial activation in the NTS. The minocycline-infused group also exhibited fewer morphological changes and less Iba-1 expression than the LPS group. Moreover, the expression of CD11b was significantly suppressed in the LPS with minocycline (L+mino) group (Fig. 3).

### Pentoxifylline effectively relieved DNA double-strand breaks in NTS mitochondria under systemic LPS infusion

**Fig. 4:**
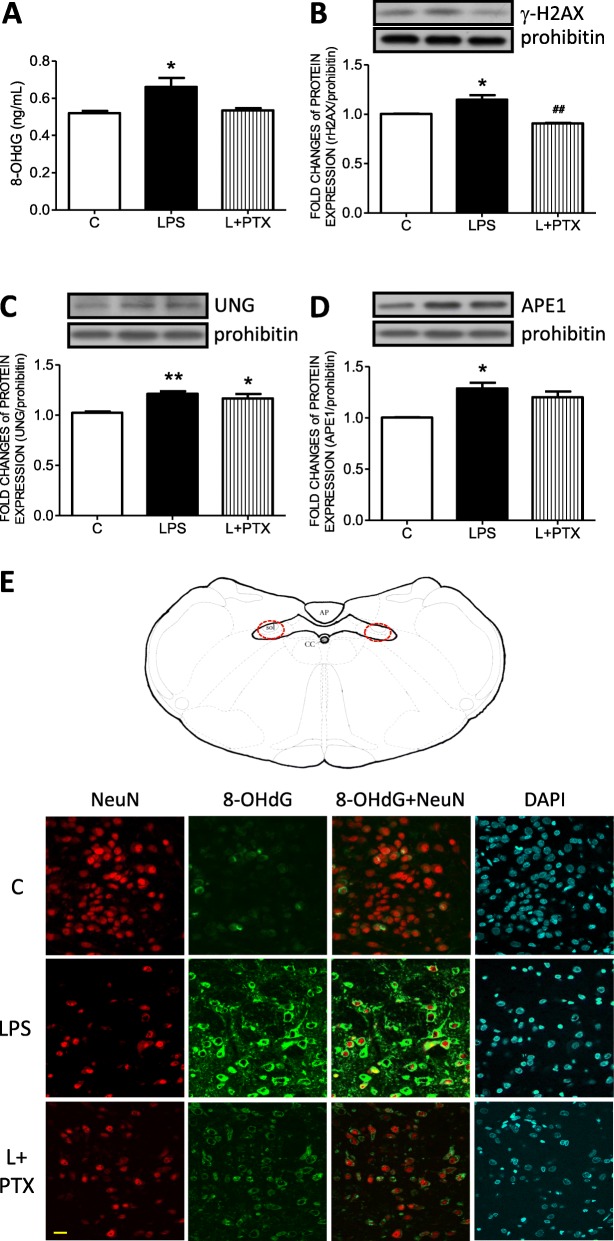
Pentoxifylline prevented mitochondrial DNA damage in the NTS induced by LPS peritoneal infusion. **a** The mitochondrial 8-OHdG levels detected by ELISA and representative gels (inset) and densitometric analysis from Western blot showed the mitochondrial protein expression of **b** γ-H2AX, **c** UNG, and **d** APE1 in the NTS after peritoneal infusion with saline or LPS for 7 days with additional intracisternal (IC) infusion of saline or PTX. Prohibitin was used as the internal control for mitochondrial protein expression. **e** A representative schematic diagram and microscopy images of 8-OHdG (green), NeuN (red), and DAPI (blue) triple immunofluorescent staining in the NTS. Values are the mean ± SEM of 6 to 8 animals in each group. *P < 0.05, **P < 0.01 versus the control (C) group and ^*##*^*P* < 0.01 versus the LPS group according to post hoc Tukey’s multiple range analysis. AP, area postrema; APE1, the apurinic/apyrimidinic endonuclease; C, control, infused with saline; cc, central canal; ELISA, enzyme-linked immunosorbent assay; L, LPS, lipopolysaccharides; PTX, pentoxifylline; sol, solitary tract; UNG, uracil-DNA glycosylase. Scale bar, 20 μm

To evaluate whether mtDNA was damaged under neuroinflammation, the level of 8-OHdG, an index of DNA oxidative damage, was detected by ELISA. The results indicated an increase in 8-OHdG in the NTS of the LPS group (Fig. 4a). Moreover, the expression of γ-H2AX (an index of DNA double-strand breaks) in the mitochondrial fraction was significantly increased in the LPS group (Fig. 4b), while no significant differences in γ-H2AX levels were detected in the nuclear fraction (Additional file 1: Figure S1).

To further evaluate the role of cytokines in the accumulation of mtDNA damage in the NTS, PTX was intracisternally (IC) infused into the NTS. The results indicated that the enhanced levels of both 8-OHdG and γ-H2AX were effectively reversed by PTX (Fig. 4).

To examine whether the BER system in mitochondria is involved in mtDNA repair, the expression levels of mitochondrial UNG and APE1 were detected by Western blot. The results indicated that UNG (Fig. 4c) and APE1 (Fig. 4d) were increased in the LPS group, while PTX application did not inhibit the enhanced levels of DNA repair enzymes.

To identify the location of mtDNA damage in the NTS, a triple immunofluorescent method with 8-OHdG (green fluorescence), NeuN (red fluorescence, a neuron cell marker), and DAPI (blue fluorescence, a nuclear dye) was employed. Representative images from the red circled area of the NTS, as depicted in the schematic, further indicated that in the LPS group, 8-OHdG largely accumulated in the cytosol instead of in the nuclei in the NTS. PTX treatment effectively relieved 8-OHdG cytosolic accumulation (Fig. 4e).

### Minocycline effectively relieved DNA double-strand breaks in NTS mitochondria under systemic LPS infusion

**Fig. 5:**
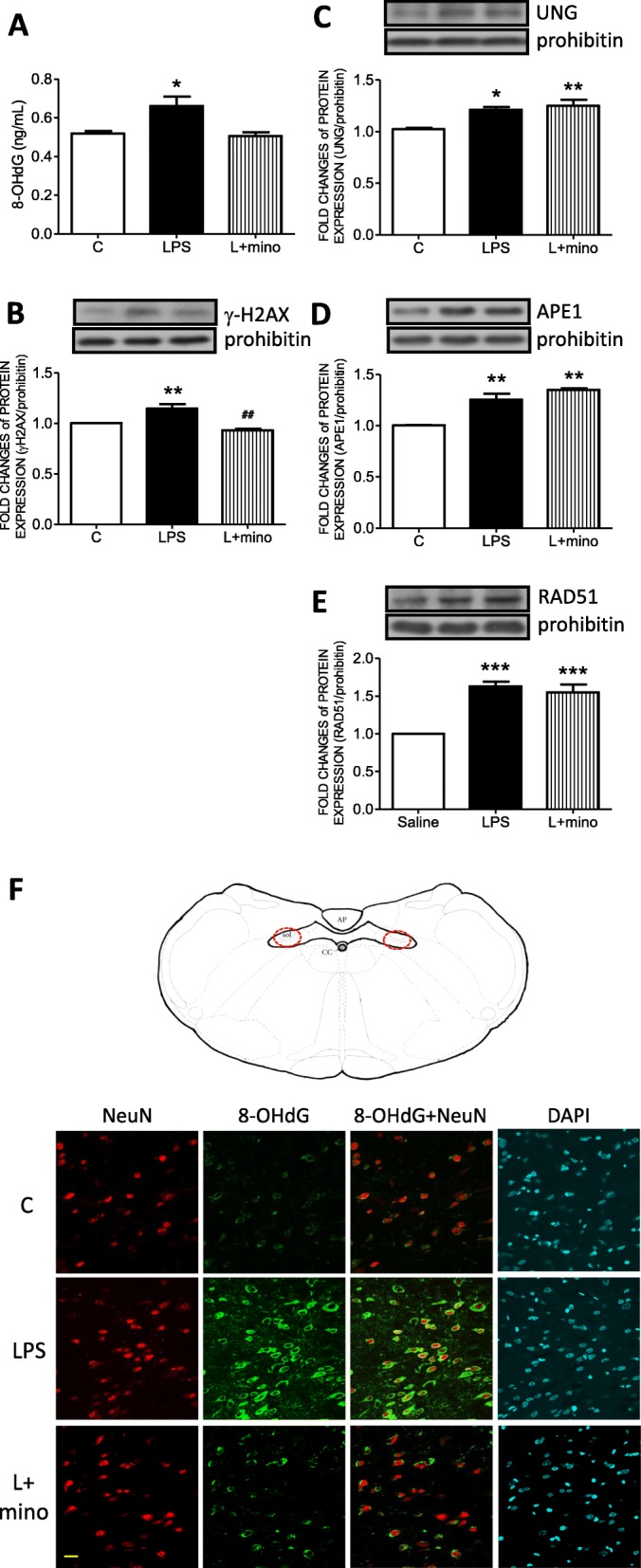
Minocycline prevented mitochondrial DNA damage in the NTS induced by LPS peritoneal infusion. **a** Mitochondrial 8-OHdG levels detected by ELISA and representative gels (inset) and densitometric analysis from Western blot showed the mitochondrial protein expression of **b** γ-H2AX, **c** UNG, **d** APE1, and **e** RAD51 in the NTS after peritoneal infusion with saline or LPS for 7 days with additional intracisternal (IC) infusion of saline or mino. Prohibitin was used as the internal control for mitochondrial protein expression. **f** A representative schematic diagram and microscopy images of 8-OHdG (green), NeuN (red), and DAPI (blue) triple immunofluorescent staining in the NTS. Values are the mean ± SEM of 6 to 8 animals in each group. *P < 0.05, **P < 0.01 versus the control (C) group and ^*##*^*P* < 0.01 versus the LPS group according to post hoc Tukey’s multiple range analysis. AP, area postrema; APE1, the apurinic/apyrimidinic endonuclease; C, control, infused with saline; cc, central canal; ELISA, enzyme-linked immunosorbent assay; L, LPS, lipopolysaccharides; mino, minocycline; sol, solitary tract; UNG, uracil-DNA glycosylase. Scale bar, 20 μm

To evaluate the role of microglial activation in the accumulation of mtDNA damage in the NTS, minocycline was IC infused into the NTS. The level of 8-OHdG was detected by ELISA. The results indicated that the increase in 8-OHdG in the NTS of the LPS group was effectively reversed by minocycline treatment (Fig. 5a). Furthermore, the increase in γ-H2AX in the mitochondrial fraction, as observed in the LPS group, was decreased in the LPS group that was additionally treated with minocycline (L+mino) (Fig. 5b).

To examine whether microglial activation triggered the BER system in mitochondria to repair mtDNA damage, the expression levels of mitochondrial UNG and APE1 were detected by Western blot. The results indicated that the increased UNG (Fig. 5c) and APE1 (Fig. 5d) by microglial activation did not inhibit the enhanced levels of DNA repair enzymes. Moreover, mitochondrial RAD51 (Fig. 5e), which is critical for the maintenance of mtDNA, was increased in the LPS group, while minocycline application did not relieve the upregulation of mitochondrial RAD51. On the other hand, no significant changes were observed in mitochondrial dynamic proteins, including FIS1 (Additional file 1: Figure S2A), p-Drp1 (Additional file 1: Figure S2B), MFN1 (Additional file 1: Figure S2C), and MFN2 (Additional file 1: Figure S2D), among the groups.

Triple immunofluorescent staining with 8-OHdG (green fluorescence), NeuN (red fluorescence, a neuron cell marker), and DAPI (blue fluorescence, a nuclear dye) was conducted in the NTS to further evaluate the effect of microglial activation on mtDNA damage. Representative images from the red circled area in the NTS, as depicted in the schematic, further indicated that 8-OHdG largely accumulated in the cytosol in the NTS of the LPS group and was effectively relieved by minocycline treatment (Fig. 5f).

## Discussion

In contrast to acute and drastic effects, our study suggested that sustained low-grade systemic inflammation triggered neuroinflammation in the NTS, resulting in suppression of the LF/HF ratio and BEI followed by increased BP. Furthermore, we report novel findings that mtDNA damage in NTS neurons mediates the neuroinflammation-impaired baroreflex sensitivity to increase BP. PTX treatment to reduce cytokine expression effectively reversed mtDNA damage, suggesting that inflammation contributed to mtDNA damage. Minocycline treatment mitigated mtDNA damage, further suggesting that microglial activation mediates mtDNA damage.

Inflammation is an important risk factor in the pathogenesis of cardiovascular diseases, as suggested by studies of both animal models and human diseases [25, 26]. Intraperitoneal LPS-induced systemic inflammation is a common model for related studies. Low-dose LPS induces hypertension [2, 4], while high-dose LPS induces drastic hypotension [27], which might be a result of different levels of cytokine release. In this study, we focused on the sustained effects of low-grade systemic inflammation on the progression of neuroinflammation. To investigate the mechanisms involved in inflammation-associated cardiovascular diseases, we developed a rodent model by intraperitoneal continuous infusion of LPS delivered via an osmotic minipump (flow rate 125 μg/h) to induce sustained low-grade systemic inflammation. This dosage is much lower than that delivered by single LPS injections [28]. Our data indicated that the evoked levels of plasma TNF-α and IL-1β in this model were lower than those reported in a previous study of high-dose LPS injection [28, 29]. Concurrent with the evoked cytokines, SBP increased with decreases in HR, the LF/HF ratio and BEI. These data implied a correlation between systemic inflammation and hemodynamic dysregulation. On the other hand, intracisternal infusion with PTX or minocycline did not significantly reduce the evoked plasma cytokines, suggesting that the pharmacological effects may be limited to the application site at these dosages.

Despite the peripheral effects of inflammation in cardiovascular disease, the central dysregulation of sympathetic and parasympathetic activities is considered the key factor of increased BP [2, 4], while the NTS negatively regulates sympathetic activity and positively controls parasympathetic tone to prevent high BP. In this study, we demonstrated that increased BP was strongly related to a decrease in the LF/HF ratio, suggesting an imbalance between sympathetic and parasympathetic activities. Suppression of the BEI further indicated baroreflex dysfunction under systemic inflammation. Growing evidence indicates that systemic inflammation can induce neuroinflammation to impair brain functions, including cardiovascular regulation [2] and cognition [30] by enhancing the release of proinflammatory cytokines from microglia [2, 4, 31]. With these lines of evidence, an intriguing question emerges: How does neuroinflammation triggered by sustained systemic inflammation dampen neuronal function?

The present study highlighted that a proinflammatory cytokine, TNF-α, in the NTS was significantly enhanced by peritoneal infusion of LPS for 7 days. TNF-α is a major cytokine that initiates chronic inflammation [32] and the onset of neurodegeneration [33, 34]. Interestingly, our data indicated that the increased TNF-α in the NTS was associated with baroreflex dysregulation and aberrant hemodynamics, which is similar to observations in Alzheimer’s disease [35] and Parkinson’s diseases [36], suggesting a link between TNF-α and neuronal dysfunction in the NTS. Due to the limitations of the study design, the involvement of TNF-α signaling in NTS dysfunction is currently unclear and requires further delineation. In contrast to the rostral ventrolateral medulla [2] and the paraventricular nucleus [26], IL-1β and IL-6 expression was not significantly altered in the NTS. TNF-α, IL-6, and IL-1β are not always concurrently upregulated, although they are all proinflammatory cytokines. For example, TNF-α [37] and IL-1β [38] have been documented to induce IL-6 expression, while IL-1β induces TNF-α expression [39]. The data in this study indicated in the NTS, only TNF-α was upregulated in the LPS group. Following this line of evidence, we reasoned that the upregulation of IL-1β and IL-6 might be detected if we checked the time point earlier and later than day 7 after LPS infusion, respectively. On the other hand, the expression of TNF-α, IL-6, and IL-1β could be region specific in different brain areas. According to Bossù et al. [40], TNF-α upregulation only occurred in the frontal cortex and the hippocampus but not in the cerebellum, the striatum or the hypothalamus on day 7 after intraperitoneal injection with LPS. Our previous study indicated that TNF-α, IL-6, and IL-1β were upregulated in the rostral ventrolateral medulla within 14 days of intraperitoneal injection with LPS [2]. We emphasize in this study that mtDNA was damaged with the mild increase in TNF-α. Based on these results, it is possible that mtDNA is damaged as early as at the initiation of proinflammatory cytokine release. The sustained, accumulated mtDNA damage in neurons could dampen neuronal functions if the damaged mtDNA in NTS neurons has not been properly repaired.

TNF-α induces DNA damage [41, 42] by increasing mitochondrial reactive oxygen species (mtROS) [43]. mtDNAs encode 13 respiratory chain proteins, tRNAs and rRNAs, which are needed for mitochondrial respiration and translation [44]. mtDNAs are a small, double-stranded circular molecules in the mitochondrial matrix that are located close to the source of mtROS concurrent with the lack of histone protection, making mtDNA the main targets of oxidative damage [45]. Moreover, the lack of nucleotide excision repair in mitochondria makes mtDNA repair inefficient compared with nuclear DNA repair [46–48]. Indeed, our study demonstrated that mtDNA oxidative damage in the NTS was significantly increased in the LPS group. The accumulation of mtDNA damage in the brain has been linked to the development of Alzheimer’s disease, Parkinson’s disease, and amyotrophic lateral sclerosis [49]. In cardiovascular regulation, we demonstrated that mtDNA accumulation in NTS neurons contributed to impaired baroreflex effectiveness, resulting in increased BP. Intracisternal infusion with PTX to inhibit TNF-α expression effectively protected neurons in the NTS from mtDNA damage. These results suggested that TNF-α mediated mtDNA damage in NTS neurons. Furthermore, intracisternal infusion with minocycline to inhibit microglial activation effectively protected NTS neurons from mtDNA damage, suggesting that microglial activation mediated mtDNA damage in NTS neurons. Moreover, these interventions effectively reversed the baroreflex desensitization and the pressor response. These results further suggested that neuroinflammation-induced mtDNA damage in NTS neurons contributed to baroreflex dysregulation. Several studies have demonstrated that mitochondrial dysfunction is a key factor involved in the central dysregulation of hemodynamics [2, 4]. Our study further highlighted the role of neuronal mtDNA damage induced by neuroinflammation in baroreflex dysregulation.

Mitochondrial dynamics play an important role in removing impaired mitochondria to maintain mitochondrial homeostasis. However, the protein expression of mitochondrial fission 1 protein (FIS1), phospho-dynamin related protein 1 (p-Drp1), mitofusin 1 (MFN1), and MFN2 in mitochondrial fractions showed no significant alterations among groups. These results suggested that mitochondrial dynamics might not be sensitive to this level of mtDNA damage in NTS neurons.

Although BER is the only mechanism for the repair of mtDNA damage [50], the results from Western blotting indicated that the expression of mitochondrial UNG, APE1, and RAD51 in the NTS was significantly enhanced in response to the neuroinflammation-induced mtDNA damage. These results suggested that the BER mechanism was activated in response to damage due to neuroinflammation. When the amount of damaged mtDNA exceeds the amount of mtDNA repaired, the neuron becomes dysfunctional. PTX or minocycline treatment mitigated the induced neuroinflammation and promoted the BER function so that the baroreflex sensitivity and hemodynamics could be properly reversed. PTX or minocycline was applied to relieve cytokine accumulation and microglial activation, respectively. Both cytokines and microglial activation (the indices of neuroinflammation) lead to oxidative stress, which is strongly bound to the oxidative damage of mtDNA. Our data further indicated that PTX or minocycline treatment not only relieved neuroinflammation but also maintained BER signaling at high levels, implying suppression of BER signaling by mtROS at the physiological level even though BER signaling is the only repair pathway in response to mtDNA damage.

## Conclusion

We demonstrated that systemic inflammation induced neuroinflammation in the NTS in this study. The neuroinflammation-triggered neuronal mtDNA damage contributed to baroreflex desensitization, while anti-inflammatory treatments not only mitigated neuroinflammation but also maintained the levels of BER function to reverse baroreflex regulation. According to these results, mtDNA could be a potential target for therapeutic strategies aimed to treat autonomic dysregulation-associated cardiovascular diseases.

## Supplementary information

**Additional file 1:** **Figure S1.** The levels of DNA double-strand breaks of nuclear DNA in NTS. The enzyme-linked immunosorbent assay (ELISA) of nuclear 8-OHdG showed no significant difference in NTS after peritoneal infusion with saline or LPS for 1 week with additional intracisternal (IC) infusion of saline. Values are mean ± SEM (*n* = 6) in the Student *t* tests. LPS: lipopolysaccharides. **Figure S2.** The representative gels (inset) and densitometric analysis from Western blot showed the mitochondrial protein expressions of (A) FIS1, (B) p-Drp1, (C) MFN1 and (D) MFN2 in NTS after peritoneal infusion with saline or LPS for 7 days with additional intracisternal (IC) infusion of saline or mino. Prohibitin was used as the internal control for mitochondrial protein expression. Values are mean ± SEM of 4 to 8 animals in each group. FIS1: mitochondrial fission 1 protein; p-Drp1: phospho-dynamin related protein 1; MFN: mitofusin; L: LPS, lipopolysaccharides; mino: minocycline.

## Data Availability

All data generated or analyzed during this study are included in this article.

## Abbreviations

APE1: Apurinic/apyrimidinic endonuclease 1
GAPDH: Glyceraldehyde 3-phosphate dehydrogenase
HF: High-frequency element of power spectral analysis from systolic blood pressure
HR: Heart rate
IC: Intracisternal
IL-1β: Interleukin-1β
LF: Low-frequency element of power spectral analysis from systolic blood pressure
LPS: *E. coli* lipopolysaccharide
mino: Minocycline
mtDNA: Mitochondrial DNA
NTS: Nucleus of the solitary tract
PTX: Pentoxifylline
RAD51: DNA repair protein RAD51 homolog 1
ROS: Reactive oxygen species
SBP: Systolic blood pressure
TBP: TATA-binding protein
UNG: Uracil-DNA glycosylase
