# Supplementary material for: Anti-neuroinflammation ameliorates systemic inflammation-induced mitochondrial DNA impairment in the nucleus of the solitary tract and cardiovascular reflex dysfunction
Source: J Neuroinflammation. 2019 Nov 15;16:224. doi: 10.1186/s12974-019-1623-0 (PMC6858639; doi:10.1186/s12974-019-1623-0)
Supplement: Supplementary file 1 — Additional file 1: Figure S1. The levels of DNA double-strand breaks of nuclear DNA in NTS. The enzyme-linked immunosorbent assay (ELISA) of nuclear 8-OHdG showed no significant difference in NTS after peritoneal infusion with saline or LPS for 1 week with additional intracisternal (IC) infusion of saline. Values are mean ± SEM (n = 6) in the Student t tests. LPS: lipopolysaccharides. Figure S2. The representative gels (inset) and densitometric analysis from Western blot showed the mitochondrial protein expressions of (A) FIS1, (B) p-Drp1, (C) MFN1 and (D) MFN2 in NTS after peritoneal infusion with saline or LPS for 7 days with additional intracisternal (IC) infusion of saline or mino. Prohibitin was used as the internal control for mitochondrial protein expression. Values are mean ± SEM of 4 to 8 animals in each group. FIS1: mitochondrial fission 1 protein; p-Drp1: phospho-dynamin related protein 1; MFN: mitofusin; L: LPS, lipopolysaccharides; mino: minocycline. [file 12974_2019_1623_MOESM1_ESM.pdf]

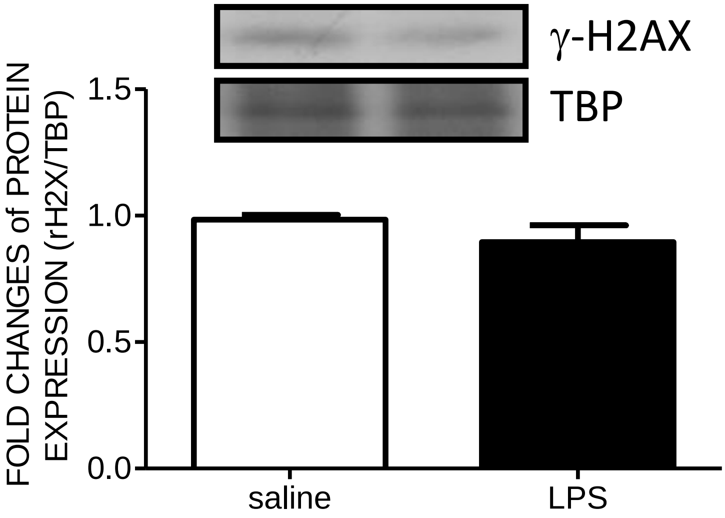

Suppl. 1. The levels of DNA double-strand breaks of nuclear DNA in NTS. The enzyme-linked immunosorbent assay (ELISA) of nuclear 8-OHdG showed no significant difference in NTS after peritoneal infusion with saline or LPS for 1 week with additional intracisternal (IC) infusion of saline. Values are mean ± SEM (n = 6) in the Student *t* tests. LPS: lipopolysaccharides.

Supplemental Fig. S1

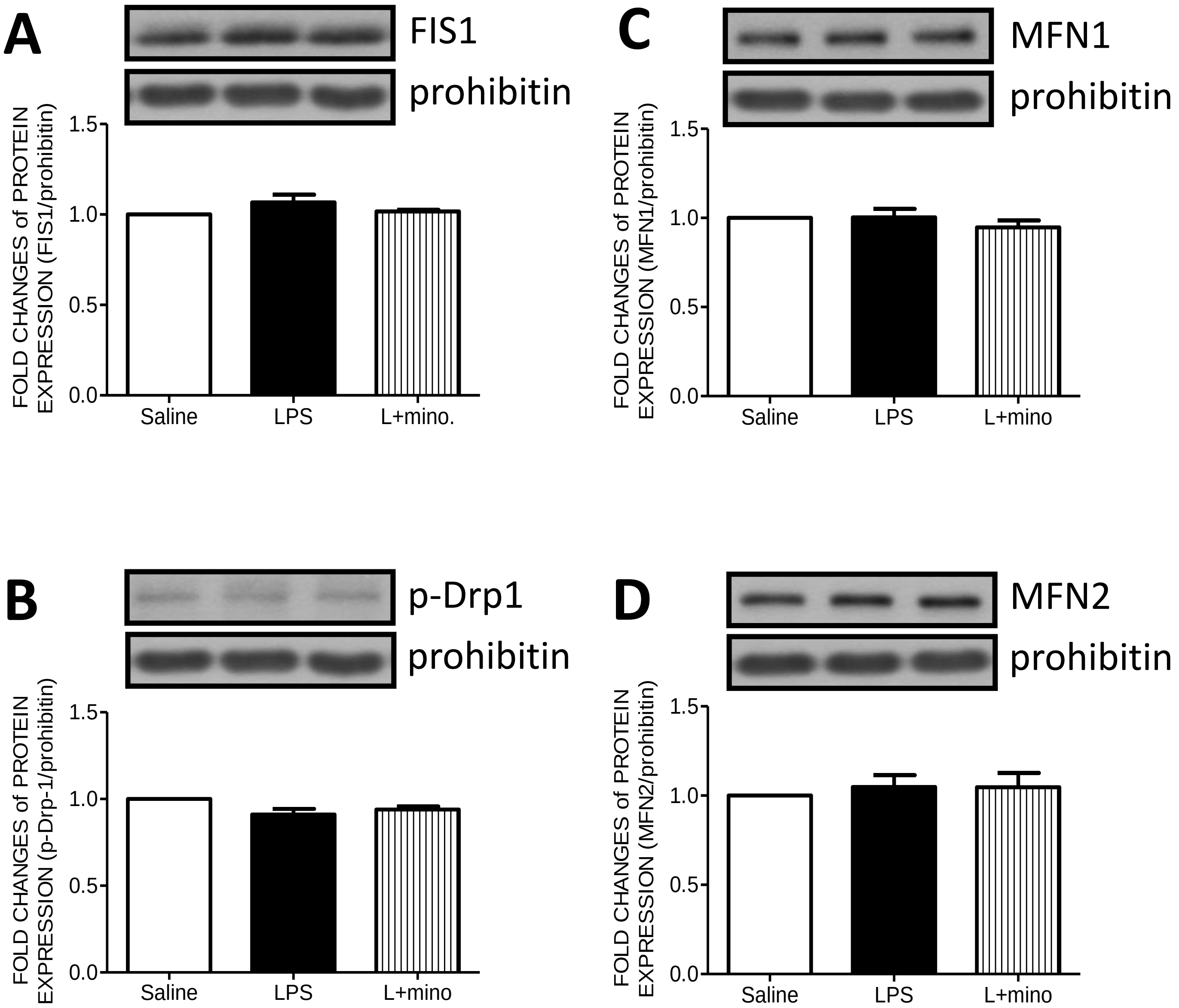

Suppl. 2. The representative gels (inset) and densitometric analysis from Western blot showed the mitochondrial protein expressions of (A) FIS1, (B) p-Drp1, (C) MFN1 and (D) MFN2 in NTS after peritoneal infusion with saline or LPS for 7 days with additional intracisternal (IC) infusion of saline or mino. Prohibitin was used as the internal control for mitochondrial protein expression. Values are mean ± SEM of 4 to 8 animals in each group. FIS1: mitochondrial fission 1 protein; p-Drp1: phospho-dynamin related protein 1; MFN: mitofusin; L: LPS, lipopolysaccharides; mino: minocycline.

Supplemental Fig. S2
